# Approximate Solutions of the Nonlinear Schrödinger Equation for Ground and Excited States of Bose-Einstein Condensates

**DOI:** 10.6028/jres.101.054

**Published:** 1996

**Authors:** R. J. Dodd

**Affiliations:** National Institute of Standards and Technology, Gaithersburg, MD 20899-0001; Institute for Physical Science and Technology, University of Maryland at College Park, College Park, MD 20742

**Keywords:** Bose-Einstein condensation, Ginzburg-Pitaevskii-Gross energy functional, Gross-Pitaevskii equation, mean-field theory, superfluidity, Thomas-Fermi functional model, time-independent nonlinear Schrödinger equation, variational wave function, vortex formation

## Abstract

I present simple analytical methods for computing the properties of ground and excited states of Bose-Einstein condensates, and compare their results to extensive numerical simulations. I consider the effect of vortices in the condensate for both positive and negative scattering lengths, *a*, and find an analytical expression for the large-*N*_0_ limit of the vortex critical frequency for *a* > 0, by approximate solution of the time-independent nonlinear Schrödinger equation.

## 1. Introduction

The recent production of trapped atomic vapors of ^87^Rb [1], ^7^Li [2], and ^23^Na [3] at the phase space densities necessary to generate Bose-Einstein condensation (BEC) in the ground state of magnetic traps has generated significant interest in this area of physics. Previously, BEC has only been encountered in the He II phase of liquid ^4^He, and there has also been accumulating evidence for BEC of an exciton gas in cuprous oxide [4]. However, it now seems that the fraction of atoms that participate in BEC in He II is of the order of 10 %, because of the presence of strong atomic interactions [5, 6]. In the dilute alkali gas BECs, such interactions are much weaker, and experimental techniques have been used to produce samples in which the condensate fraction is very close to unity. This makes the alkali systems most attractive for the study of the essential phenomena of BEC, especially since the condensate density and temperature, and perhaps even the intrinsic atomic interactions [7], are subject to external experimental control.

However, the alkali condensates are always produced in particle traps, and this endows them with an essentially inhomogeneous character quite distinct from the homogensous nature of ^4^He II. Detailed understanding of the properties of these systems requires [8–16] that this inhomogeneity be taken into account. The purpose of this paper is to present simple analytical methods which have been found to give results that are in good qualitative agreement with those of extensive numerical calculations based on mean-field theory, i.e., the basis-set methods of Refs. [12, 16]. These methods are thus expected to be useful to experimentalists who wish to explore alternative trap designs, or to modellers who wish to analyze large-scale numerical results.

## 2. Overview of Dilute Gas Bose-Einstein Condensates

For ^87^Rb and ^23^Na the scattering lengths, *a*, are known to be positive [3,17,18]. In standard mean-field theory, this leads to a stable condensate wave function which is larger than the noninteracting ground state of the trap, and which can be modeled well by a mean field equation [8–16]. For ^7^Li, *a* is known to be negative [2], which produces a net effective attractive interaction between the particles. A homogeneous condensate with *a* < 0 is predicted to be unstable [19]. However, a spatially confined condensate has been shown to be metastable [9]; and, when the average number of atoms in the condensate, *N*_0_, is sufficiently small, the condensate lifetime can be quite long. The potential energy of the confined system acts as a barrier to the collapse of the condensate [9, 11]. Other discussions of the negative scattering length case have addressed the general issues of energetic stability and the possibility of a transition to a denser phase [20–22]. Theoretical predictions of the sizes and lifetimes of Bose condensed gases can now be compared directly with experiments, as will be discussed below. The effect of vortex formation [11–14] may also be examined in the present framework.

In this paper, I examine the general cases of the two approximate methods which have been most used to study Bose condensed systems, i.e., the Thomas-Fermi model [8, 11], and the variational method [11]. While the ground state properties of such Bose condensed gases have been studied in some detail [8–16], most of the methods used have been computationally intensive. I show here that approximate methods give good quantitative results for the ground state properties of these gases, and also for vortex solutions of the equations of motion.

## 3. Basic Results of Mean-Field Theory

For the low atomic energies and densities achieved in the experiments of Refs. [1–3] the basic structure of a BEC should be well approximated by mean field theory, i.e., the Ginzburg-Pitaevskii-Gross (GPG) energy functional [23], described in Eq. (1). In such cases the atom-atom interaction is dominated by the effects of *s*-wave collisions. The interaction potential may thus be encapsulated in the form *V*(***r***, ***r***′) = *U*_0_*δ* (***r***–***r***′), where *U*_0_ = 4*πħ*^2^*a*/*M*. Solutions of the resulting many-particle mean-field Schrödinger equation provide information about the size, density, chemical potential, and lifetime of the condensate as a function of *N*_0_. The evolution of a condensate, after release from the weak trap of Ref. [1] for time of flight studies, has been described using the time-dependent solutions of the Gross-Pitaevskii (GP) equation in Ref. [10]. Here I consider the condensates formed in the tight trap [1], which should be adequately represented by a time-independent formalism. The Gross-Pitaevskii-Gross (GPG) energy functional [23] for this system may be written

$$\begin{array}{c} \varepsilon \left[\psi \right]=\int dr\;\psi *\left(r\right)\\ \left(-\frac{\hbar^{2}}{2M}\nabla^{2}+V_{\text{trap}}\left(r\right)+\frac{1}{2}N_{0}U_{0}{\left|\psi \left(r\right)\right|}^{2}\right)\psi \left(r\right), \end{array}$$ (1)

where *ψ*(***r***) is the common wavefunction for each atom in the condensate, and *ε*[*ψ*] is the energy per particle in the system. Minimizing *ε*[*ψ*] with respect to variations in the wavefunction *ψ*(***r***), while enforcing the normalization condition, ∫d***r***|*ψ*(***r***)|^2^ = 1, gives the time-independent GPG equation [23].

$$\begin{array}{c} \left(-\frac{\hbar^{2}}{2M}\nabla^{2}+V_{\text{trap}}\left(r\right)+N_{0}U_{0}{\left|\psi \left(r\right)\right|}^{2}\right)\\ \psi \left(r\right)=\mu \psi \left(r\right), \end{array}$$ (2)

where *V*_trap_(***r***) is usually an anisotropic harmonic oscillator potential [1, 2]. Baym and Pethick [11] have identified the dimensionless parameter for a cylindrically symmetric harmonic system as

$$\zeta ={\left(8\pi aN_{0}\alpha_{\text{trap}}\right)}^{1/5},$$ (3)

where *α*_trap_ = (*Mω_ρ_*/*ħ*)^1/2^, and $V_{\text{trap}}\left(r\right)=M\left(\omega_{\rho }^{2}\rho^{2}+\omega_{z}^{2}z^{2}\right)/2$. I use Eq. (1) to model a condensate using a variational trial wave function, and in the limit of sufficiently large *ζ* we will also examine the Thomas-Fermi model, using Eq. (2), where the kinetic energy of the condensate atoms is neglected.

An important property, of both current and future experimental interest, is the lifetime of the condensate with respect to population loss from the trapped state. Such losses derive from spin-flip collisions that force the spin-flipped atoms out of the trap. These collisions are considered here to be extrinsic to the kinetics of BEC formation, and are treated by a simple rate-equation approach. The two-body population loss rate for a condensate is given by Eq. (20) in Ref. [24]:

$$\begin{array}{c} R^{\left(2\right)}\left(N_{0}\right)=2\alpha \int dr\\ \left(\frac{1}{2}n_{0}^{2}\left(r\right)+2n_{0}\left(r\right)n^{\prime }\left(r\right)+{n^{\prime }}^{2}\left(r\right)\right), \end{array}$$ (4)

where *n*_0_(***r***) and *n*′(***r***) are the respective condensate and noncondensate number densities, and *α* is the two-body loss rate coefficient associated with spin-flip collisions. Since, by the use of forced evaporative cooling, the noncondensate atoms may be “cut” away [1], forcing *n*′(***r***) ~ 0, Eq. [4] reduces to

$$R^{\left(2\right)}\left(N_{0}\right)=\alpha \int dr\;n_{0}^{2}\left(r\right)=\alpha N_{0}^{2}\int dr{\left|\psi \left(r\right)\right|}^{4}.$$ (5)

Finding a solution of the GP equation, or minimum of the GPG energy functional, together with the loss-rate expression, enables one to estimate the condensate lifetime in units of *α* [16, 25].

While the ground-state properties of BECs are of primary interest, the possibility of vortex formation has also been considered [11–14]. A steady-state vortex wave function may be written as [26]

$$\psi \left(r\right)=\rho^{1/2}\left(r\right)e^{is\left(r\right)},$$ (6)

where *ρ*(***r***) is the density of the system. Here, *S*(***r***), the velocity potential, changes by 2*πm* along any closed loop around the vortex, with *m* being the vortex winding number. For the cylindrically symmetric harmonic traps of Refs. [1, 2], a wavefunction of the form in Eq. (6), with the *z*-axis as the vortex line, is related to states of definite *z*-component of the total angular momentum. The corresponding eigenfunction may be written as

$$\psi \left(r\right)=f_{n_{\rho }n_{z}m}\left(r\right)e^{im\phi },$$ (7)

where {*n_ρ_*,*n_z_*,*m*} are the radial, axial, and angular momentum (*z*-component) quantum numbers. Equation (7) is equivalent to a winding number *m* vortex state. However, for an interacting system, the function $f_{n_{\rho }n_{z}m}\left(r\right)$ will also be a function of *N*_0_. As pointed out in Refs. [11–14], a rotating system should cause the formation of a vortex line or ring with critical rotation frequency, *ν*_crit_. In a homogeneous rotating system, the critical frequency is given by [26]

$$\nu_{\text{crit}}=\frac{\hbar }{2\pi MR^{2}}\;\ln\;\left(\frac{R}{\xi }\right),$$ (8)

where *R* is the radius of the system, and *ξ* the radius of the vortex core. The critical frequency may be calculated directly from the equation D*E* – ***L***·***ω*** = 0, where Δ*E* is the energy gap between ground and vortex states, ***L*** is the vortex angular momentum, and ***ω*** is the rotational angular velocity of the system [26]. In the case of a cylindrically symmetric harmonic system, *L* = *N*_0_*mħ*, and Δ*E* = *N*_0_(*ε_m_* – *ε*_0_), where *ε_m_* is the energy per particle in the *m*th vortex state, and it is a function of *N*_0_. This gives a critical frequency of

$$\nu_{\text{crit}}=\frac{\varepsilon_{m}-\varepsilon_{0}}{2\pi m\hbar }$$ (9)

Solving the GPG equation, and finding the energy per particle of the condensate, one may calculate the critical frequency for formation of such vortices in the system.

## 4. Simplified Variational Model

For a noninteracting cylindrically symmetric harmonic system, the eigenfunctions of the Schrödinger equation (Eq. (2) with *U*_0_ = 0) are

$$\begin{array}{r} \psi_{n_{\rho }n_{z}m}(r)={\left[\frac{\alpha_{z}}{\pi^{1/2}2^{n_{z}}n_{z}!}\right]}^{1/2}e^{-\alpha_{z}^{2}z^{2}/2}H_{n_{z}}(\alpha_{z}z)\\ \times {\left[\frac{\alpha_{\rho }^{2}n_{\rho }!}{\pi (n_{\rho }+m)!}\right]}^{1/2}e^{im\phi }(\alpha_{\rho }\rho {)}^{m}e^{-\alpha_{\rho }^{2}\rho^{2}/2}L_{n_{\rho }}^{\left(m\right)}(\alpha_{\rho }^{2}\rho^{2}), \end{array}$$ (10)

where *α_ρ_*_,z_ = (*MΩ_ρ_*_,z_/*ħ*)^1/2^, *H_n_*(*x*) is the *n*-th order Hermite polynomial, and *L_n_*^(^*^m^*^)^ (*x*) is the *m*-th associated Laguerre polynomial of order *n* [27]. In the noninteracting case (i.e., *U*_0_ = 0) *Ω_ρ_* = *ω_ρ_* and *Ω_z_* = *ω*_z_, however, using Eqs. (10) as variational trial wavefunctions for the interacting system, with *Ω*_ρ_ and *Ω_z_* treated as arbitrary variational parameters, we find the GPG energy functional,

$$\begin{array}{c} \varepsilon [\Omega_{\rho },\Omega_{z}]=\frac{\hbar }{2}[(2n_{\rho }+m+1)\;\left(\Omega_{\rho }+\frac{\omega_{\rho }^{2}}{\Omega_{\rho }}\right)+\\ \left(n_{z}+\frac{1}{2}\right)\;\left(\Omega_{z}+\frac{\omega_{z}^{2}}{\Omega_{z}}\right)+\frac{\pi \zeta^{5}}{{\left(2\pi \right)}^{5/2}}\;\frac{\Omega_{\rho }}{\omega_{\rho }^{1/2}}\;\Omega_{z}^{1/2}\;\beta_{n_{\rho }n_{z}m}], \end{array}$$ (11)

where

$$\begin{array}{c} \beta_{n_{\rho }n_{z}m}=2\sqrt{\frac{2}{\pi }}\;{\left[\frac{n_{\rho }!}{2^{n_{z}}n_{z}!(n_{\rho }+m)!}\right]}^{2}\\ \int_{-\infty }^{\infty }dve^{-2v^{2}}|H_{n_{z}}(v){|}^{4}\int_{0}^{\infty }due^{-2u}u^{2m}|L_{n_{\rho }}^{\left(m\right)}(u){|}^{4}, \end{array}$$ (12)

is a dimensionless constant depending on the state being considered, normalized so *β*_000_ = 1, and

$$\beta_{0\;0m}=\frac{(2m)!}{2^{2m}{\left(m!\right)}^{2}}$$ (13)

is important for analysis of vortex states. While Eq. (11) is simple to analyze numerically, as I show later, it may also be used to give approximate solutions in the limit of *ζ* ≫ 1. While it is easy to examine the perturbative limit, |*ζ*l ≪ 1, it is of little experimental interest, since |*ζ*| ≥ 1 in all experiments to date [1].

I minimize Eq. (11) with respect to the variational parameters, *Ω_ρ_* and *Ω_z_*, to find,

$$\begin{array}{c} 2(2\pi {)}^{5/2}(n_{z}+1/2)\Omega_{z}^{1/2}\omega_{\rho }^{1/2}(\Omega_{z}^{2}-\omega_{z}^{2})\\ +\pi \beta_{n_{\rho }n_{z}m}\zeta^{5}\Omega_{\rho }\Omega_{z}^{2}=0, \end{array}$$ (14)

given *Ω*_ρ_ = *ω_ρ_*/***Δ***, found from ∂*ε*/∂*Ω_ρ_* = 0, where one has

$$\Delta ={\left[1+\frac{\zeta^{5}}{{\left(32\pi \right)}^{1/2}}{\left(\frac{\Omega_{z}}{\omega_{\rho }}\right)}^{1/2}\frac{\beta_{n_{\rho }n_{z}m}}{\left(2n_{\rho }+m+1\right)}\right]}^{1/2}.$$ (15)

When *N*_0_ is sufficiently large, *Ω_ρ_* ≪ *ω*_ρ_ and *Ω_z_* ≪ *ω*_z_, so one needs only to examine terms of highest-order in *ω_ρ_* and *ω*_z_. This is directly equivalent to neglecting the kinetic-energy terms in Eq. (11). This approximation then gives

$$\Omega_{z}=\omega_{z}{\left[\frac{{\left(2n_{z}+1\right)}^{4}}{{\left(2n_{\rho }+m+1\right)}^{2}}\frac{\omega_{z}^{3}}{\omega_{\rho }^{3}}\frac{{\left(2\pi \right)}^{3}}{\zeta^{10}\beta_{n_{\rho }n_{z}m}^{2}}\right]}^{1/5}$$ (16)

$$={\left[\frac{{\left(2n_{z}+1\right)}^{4}}{{\left(2n_{\rho }+m+1\right)}^{2}}\frac{\omega_{z}^{8}}{\omega_{\rho }^{4}}\frac{\pi \hbar }{8Ma^{2}N_{0}^{2}\beta_{n_{\rho }n_{z}m}^{2}}\right]}^{1/5}$$ (17)

and

$$\Omega_{\rho }=\omega_{\rho }{\left[\frac{{\left(2n_{\rho }+m+1\right)}^{3}}{\left(2n_{z}+1\right)}\frac{\omega_{\rho }^{2}}{\omega_{z}^{2}}\frac{{\left(2\pi \right)}^{3}}{\zeta^{10}\beta_{n_{\rho }n_{z}m}}\right]}^{1/5}$$ (18)

$$={\left[\frac{{\left(2n_{\rho }+m+1\right)}^{3}}{\left(2n_{z}+1\right)}\frac{\omega_{\rho }^{6}}{\omega_{z}^{2}}\frac{\pi \hbar }{8Ma^{2}N_{0}^{2}\beta_{n_{\rho }n_{z}m}^{2}}\right]}^{1/5}.$$ (19)

These expressions may be substituted back into Eq. (11) in order to evaluate the energy per particle of the system, and one may then calculate the critical frequency for vortex formation. For *a* > 0, the lowest vortex critical frequency occurs for the ground state to {*n_ρ_* = 0, *n_z_* = 0, *m* = 1} transition, giving

$$\begin{array}{c} v_{\text{crit}}=\frac{3}{4\pi }{\Omega_{\rho }}^{\left\{n_{\rho }=0,n_{z}=0,m=0\right\}}\sim {\left(\frac{\omega_{z}^{2}}{\omega_{\rho }}\right)}^{4/5}\zeta^{-2},\\ =\frac{3\hbar }{4\pi M\rho_{1/e}^{2}}, \end{array}$$ (20)

where *ρ*_1/e_ is the radius of the 1/e population density of the ground-state condensate in the *z* = 0 plane. Unlike the homogeneous case of ^4^He II, where *ν*_crit_ ~ ln(*R*/*ξ*)/*R*^2^ from Eq. (8), the trapped condensate has *ν*_crit_ ~ 1/*R*^2^ in the large *N*_0_ limit, where *R* = *ρ*_1/e_ is a characteristic size of the condensate in the plane of rotation.

**Fig. 1:**
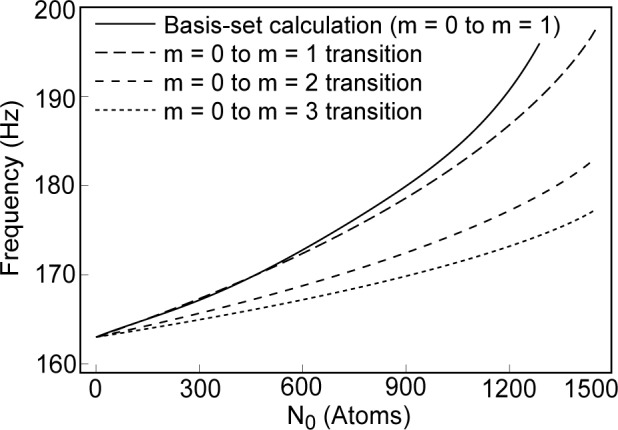
A plot of the vortex transition frequency, in a trap of frequencies *ν*_ρ_ = 163 Hz and *ν*_z_ = 117 Hz. The frequencies, calculated by the variational method, for *m* = 0 to *m* = 1 (long-dashed line), *m* = 0 to *m* = 2 (medium-dashed line), and *m* = 0 to *m* = 3 (short-dashed line) transitions to occur. The frequency for a *m* = 0 to *m* = 1 transition calculated from the basis-set expansion (solid line).

For *a* < 0, the kinetic energy is never negligible, and becomes more important as the condensate population approaches the point of collapse. Since the kinetic energy may not be neglected in comparison with the self-interaction energy, and the potential energy is of roughly the same order as the kinetic, the shrinkage of the condensate spatial distribution is not severe enough to make the potential energy much smaller than the kinetic energy. Hence, Eq. (14) must be solved numerically. Figure 1 shows plots, for the parameters appropriate to the experiment of Ref. [2], of the vortex critical rotation frequency calculated from numerical solution of Eq. (14) for transitions, *m* = 0 to *m* = 1 (long-dashed line), *m* = 0 to *m* = 2 (medium-dashed line), *m* = 0 to *m* = 3 (short-dashed line). Also shown is the frequency for an *m* = 0 to *m* = 1 transition (solid line), calculated by the basis-set method. As can be seen, the lowest critical frequency, and therefore the first transition to occur when the system is rotated, should be the *m* = 0 to *m* = ∞ transition. The maximum stable population for a condensate with negative scattering length is given by *Δ* = 0 from Eq. (15). Assuming *Ω_z_* ≈ *ω_z_*, this would give

$$N_{n_{\rho }n_{z}m}^{\max}=\left\{\frac{2n_{\rho }+m+1}{\beta_{n_{\rho }n_{z}m}}\right\}\;{\left(\frac{\pi \hbar }{2M\omega_{z}}\right)}^{1/2}\frac{1}{\left|a\right|}$$ (21)

$$=\left\{\frac{2n_{\rho }+m+1}{\beta_{n_{\rho }n_{z}m}}\right\}\;N_{000}^{\max}.$$ (22)

**Fig. 2:**
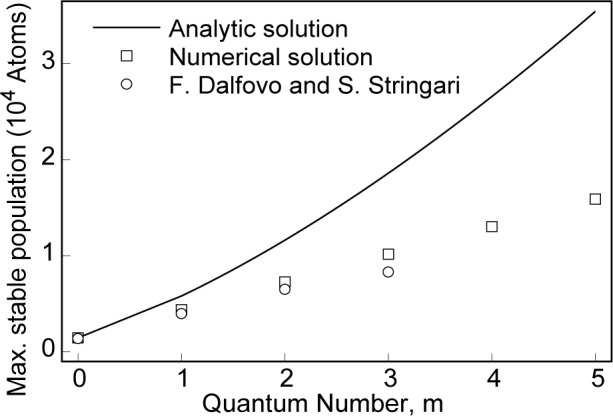
A plot of the maximum vortex population (*n_ρ_* = 0, *n_z_* = 0), the approximate analytic solution $N_{\max}^{\left\{0,0,m\right\}}=1453$ (*m* + 1)!*m*!2^2^*^m^*/(2 *m*)! (solid line), numerical solution of the minimized Ginzburg-Pitaevskii-Gross energy functional (squares), and results from [F. Dalfovo and S. Stringari, Phys. Rev. A 53, 2477 (1996)] (circles).

Equation (21) gives $N_{000}^{\max}\sim 3000$ for parameters appropriate to the experiment of Ref. [2]. However, one finds $N_{000}^{\max}\sim 1500$ by numerical solution of Eq. (14), close to the value found in Refs. [9, 12–14]. Figure 2 shows the maximum population of a vortex state in the trap of Ref. [2], comparing the results of Eq. (22) (solid line), numerical solution of Eq. (14) (squares), and results from Ref. [13] (circles). As *N*_0_ approaches the maximum stable level, many of the properties exhibit extreme behavior, i.e., the two-body loss rate has been shown to increase rapidly as the condensate population approaches the point of collapse [16].

The two-body loss rate is given, subtituting Eq. (10) into Eq. (5) by

$$R_{n_{\rho }n_{z}m}^{\left(2\right)}\;(N_{0})=\alpha N_{0}^{2}\;{\left(\frac{M}{2\pi \hbar }\right)}^{3/2}\beta_{n_{\rho }n_{z}m}\Omega_{\rho }\Omega_{z}^{1/2}.$$ (23)

**Fig. 3:**
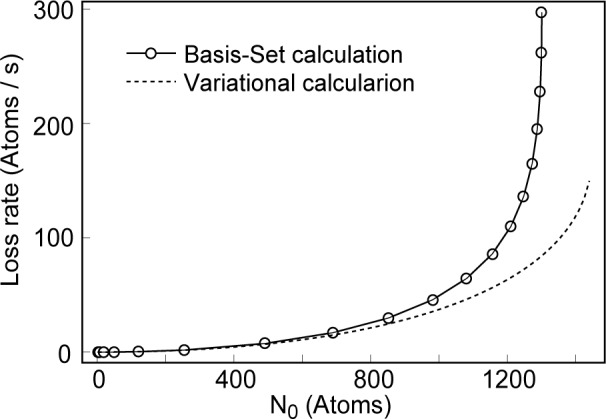
A plot of the two-body loss rate for a ^7^Li condensate in a cylindrically symmetric trap, *ν*_ρ_ = 163 Hz and *ν*_z_ = 117 Hz. Basis-set (solid line), and variational (broken line) calculations.

This shows, because $R_{n_{\rho }n_{z}m}^{\left(2\right)}\;(N_{0})<R_{n_{\rho }n_{z}(m+1)}^{\left(2\right)}\;(N_{0})$ that vortex states have longer lifetimes than those states without, or with less, angular momentum. Figure 3 shows a plot of the loss rate from a ^7^Li condensate in the experimental configuration of Ref. [2], the total loss rate [including three-body recombination (which was found to be negligible except very close to the point of collapse)] from Ref. [16] (solid line), and the two-body loss rate calculated from Eq. (23) (broken line). While the variational approach clearly does not show as rapid a growth in the loss rate as the critical population, it does demonstrate qualitatively the same behavior.

## 5. Thomas-Fermi Model

**Fig. 4:**
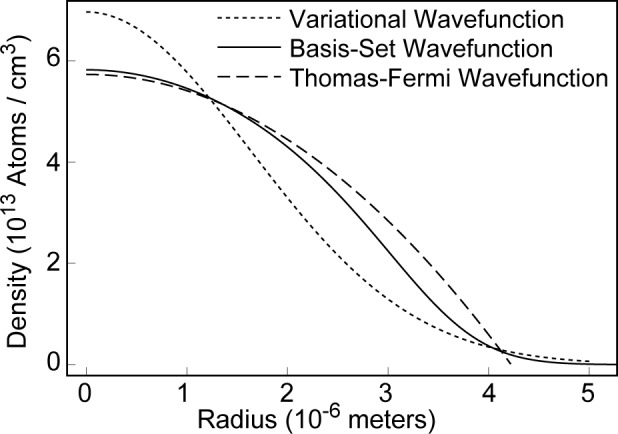
The number density of a ~ 2500 atom ^87^Rb condensate along the radial coordinate, in the *z* = 0 plane. Basis-set (solid line), variational (dotted line), and Thomas-Fermi (broken line) calculations.

**Fig. 5:**
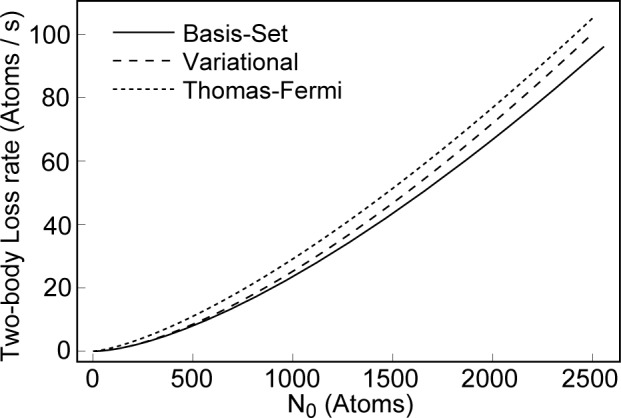
A plot of the two-body loss rate for a cylindrically symmetric ^87^Rb trap, *ν*_ρ_ = 212 Hz and *ν*_z_ = 75 Hz. Basis-set (solid line), variational (broken line), and Thomas-Fermi (dotted line) calculations. Here *α*, the two-body loss rate coefficient, is 1.3 × 10^−15^ cm^3^ s^−1^.

For a large condensate, *ξ* ≫ 1, with *a* > 0 we find

$$\langle \psi \left|-\frac{\hbar^{2}}{2M}\nabla^{2}\right|\psi \rangle \ll \langle \psi \left|N_{0}U_{0}|\psi (r){|}^{2}\;\right|\psi \rangle ,$$ (24)

meaning the kinetic energy term in Eq. (2) may be neglected [8, 11] in comparison to the condensate self-interaction energy. This of course is never true for a condensate with *a* < 0, since the kinetic energy is the stabilizing factor in preventing the collapse of the condensate to a more dense phase [9, 11]. For *a* > 0, however, this condition may be satified for a fairly small condensate population [8]. Previously, this method has been used to study ground state condensates in the large *N*_0_ limit [8, 11], but the possibility of studying vortex states has only been considered peripherally [12]. While Eq. (24) may still be true in the case of a vortex, we may not neglect the effect of the angular momentum on the spatial distribution of the condensate, since the angular momentum imposes a “centrifugal barrier.” This reduces Eq. (2) to

$$\begin{array}{c} \left(\frac{\hbar^{2}\gamma^{2}}{2M\rho^{2}}+V_{\text{trap}}\;(r)+N_{0}U_{0}|\psi (r){|}^{2}\right)\\ \psi (r)=\mu \psi (r), \end{array}$$ (25)

where the kinetic energy not related to the angular momentum has been neglected, and the angular dependence, i.e., the phase factor (which does not effect the density distribution), of the wavefunction has been ignored. For a cylindrical system one has *γ*^2^ = *m*^2^, and for a spherical system one has *γ*^2^ = *l*(*l*+1), where *l* is the total angular momentum quantum number. This gives a Thomas-Fermi wave function of the form

$$\psi (r)=\{\begin{array}{cc} {\left[\frac{\mu -V_{\text{eff}}(r)}{N_{0}N_{0}}\right]}^{1/2}, & \text{for}\;V_{\text{eff}}(r)<\mu ;\\ 0 & \text{for}\;V_{\text{eff}}(r)\geq \mu ; \end{array}$$ (26)

where *V*_eff_(***r***) = *V*_trap_(***r***) + *ħ*^2^*γ*^2^/2*Mρ* ^2^ is the effective trap potential. The relationship between *N*_0_ and *μ* is found by the conventional normalization condition, ∫d***r***|*ψ*|^2^ = 1. Figure 4 shows a plot of the number density of a 2000 atom 87Rb condensate for parameters appropriate to Ref. [1], the wave function calculated by basis-set expansion as in Ref. [12, 16] (solid line), Thomas-Fermi (brokenline), and variational (dotted line) methods. In the simplest case, a harmonic trap with *γ* = 0, many of the properties of the condensate may be calculated explicitly [8, 11], i.e., the two-body loss rate is given by

$$R^{\left(2\right)}(N_{0})=\frac{\alpha }{7}\;{\left(\frac{450}{\pi^{2}}\right)}^{1/5}\;{\left(\frac{M{\omega_{\text{trap}}}^{2}}{U_{0}}\right)}^{3/5}N_{0}^{7/5},$$ (27)

where $\omega_{\text{trap}}^{3}=\omega_{x}\omega_{y}\omega_{z}=\omega_{\rho }^{2}\omega_{z}$. Figure 5 shows the two-body loss rate from the ground state of the trap of Ref. [1], where I have assumed that *α* = 1.3 × 10^−15^ cm^3^ s^−1^ [25], comparing the results of the basis-set method (solid line), numerical evaluation of Eq. (23) (broken line), and the Thomas-Fermi expression in Eq. (27) (dotted line). Equation (5) may be integrated to give an expression for the lifetime of the condensate because of two-body collisions,

$$\tau_{1/\delta }(N_{0})=\frac{35}{2\alpha }\;{\left(\frac{\pi^{2}}{450}\right)}^{1/5}\;{\left(\frac{U_{0}}{M\omega_{\text{trap}}^{2}}\right)}^{3/5}\;(\delta^{2/5}-1)N_{0}^{-2/5},$$ (28)

where *τ*_1/*δ*_ (*N*_0_) is the 1/*δ* population lifetime of a condensate intially containing *N*_0_ atoms. This gives a 1/e population lifetime for a 2000 atom ^87^Rb condensate in the range 32 s to 280 s, for 1.5 × 10^−16^ cm^3^ s^−1^ < *α* < 1.3 × 10^−15^ cm^3^ s^−1^ [25]. This range is very close to the 35 s to 200 s reported in Ref. [12]. The longer maximum lifetime of 280 s compared to 200 s occurs because I have neglected three-body recombination, while it was considered in Ref. [12]. The condensate has a lifetime, calculated by the basis-set method, because of spin-flip collisions in the range 39 s to 340 s. As we would expect the Thomas-Fermi model gives a shorter lifetime than the basis-set method, since the condensate is more tightly confined (within a finite region of space), c.f. Fig. 4.

**Fig. 6:**
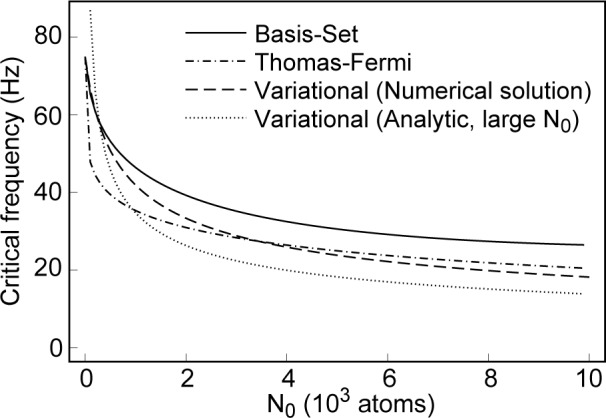
A plot of the vortex transition frequency, for *m* = 0 to *m* = 1 with a positive scattering length: the variational value calculated numerically (broken line), the variational expression for large *N*_0_ (dotted line), the Thomas-Fermi approximation (dot-dashed line), and the basis-set expansion method (solid line).

I have also calculated the vortex critical frequencies for parameters appropriate to the trap of Ref. [1] for comparison to the results obtained earlier for the variational model, and those presented in Refs. [12–14]. Figure 6 shows plots of frequencies for the *m* = 0 to *m* = 1 transition calculated from the large *N*_0_ variational expression of Eq. (20) (dotted line), the numerical solution of Eq. (14) (broken line), the basis-set expansion (solid line), and the Thomas-Fermi model (dot-dashed line). For a 10000 atom ^87^Rb condensate, in the trap of Ref. [1], I have found *ν*_crit_ = 26 Hz (basis-set expansion), 20 Hz (Thomas-Fermi), 18 Hz (numerical solution of Eqs. (14, 15), to minimize the GPG energy functional), and 14 Hz (Eq. (20), the large *N*_0_ approximation), compared to the 26 Hz reported in Ref. [13, 14]. While one would not expect the Thomas-Fermi approximation to give accurate results for relatively small *N*_0_, as can be seen from Figs. (4 and 5), there is reasonable agreement with the basis-set calculations for as few as 2000 atoms.

## 6. Conclusions

I have shown that the variational method, using the wave functions of Eq. (10), gives very good agreement with the results found by much more computationally intensive methods [8–10,12–14,16]. For *a* > 0, it is clear that the variational and Thomas-Fermi methods may be used to examine the full range of condensate populations, at least when ground states and vortex states are being considered, to provide good estimates of the condensate properties, i.e., lower bounds on lifetimes and peak densities. The Thomas-Fermi approximation also has the advantage of being analytically solvable for the most common cases of experimental interest, i.e., harmonic traps as in Refs. [1, 2]. It may also be solved numerically with little difficultly for traps of more arbitrary geometries [3]. For *a* < 0, the variational methods do not give as good agreement to the numerical calculations [13,14,16], especially as the critical population levels are approached. While qualitative behavior is retained, i.e., maximum population levels are in good agreement, other properties of the condensate, e.g., two-body collisional loss rates, do not show the same extreme behavior near the critical population levels.

The critical frequency for vortex formation in a large condensate of repulsive atoms was found to scale as *ν*_crit_ ~ 1/*R*^2^ in the limit of large *N*_0_, where *R* is the characteristic radius of the condensate in the plane of rotation, compared to a homogeneous system in which *ν*_crit_, ~ ln(*R*/*ξ*)/*R*^2^. The formation of vortices, and scaling of the vortex critical frequency, should be examined experimentally in the near future. While the approximate methods used here to calculate the vortex critical frequencies give reasonable results in comparison to the methods of Refs. [12–14], the scaling behaviour at large *N*_0_ appears to be very similar for all the methods. In the case of negative scattering length, the formation of vortices has been considered as a mechanism for forming condensates containing greater numbers of atoms [13, 14]: an *m* = 1 vortex may contain ~ 4000 atoms, compared to ~ 1300 atoms for the ground state in the trap of Ref. [2]. I have shown, however, that the formation frequency for such vortices is lowest for the *m* = ∞ state, so the actual formation of such structures in experiments must be studied more fully. Indeed a time-dependent calculation may be required to examine the condensate response to rotation near the critical vortex formation frequency. It is possible that vortices may be formed during the evaporative cooling cycle, as high energy atoms are “cut” away, and low energy atoms are ejected because of the rapid increase in loss processes [16,20–22] for lower energy states.

## Glossary

Symbol: Definition
*a*: Scattering length
*M*: Atomic mass
*μ*: Chemical potential
*N*_0_: Average number of condensate atoms
*U*_0_: Effective two-body interaction strength
*R*^(2)^(*N*_0_): Two-body spin-flip collisional loss rate
***r*** = {*ρ*, ϕ, *z*}: Cylindrical coordinate system
*ω_ρ_*, *ω_z_*: Trap radial and axial harmonic oscillator angular frequencies
